# Optilume drug‐coated balloon for anterior urethral stricture: 2‐year results of the ROBUST III trial

**DOI:** 10.1002/bco2.312

**Published:** 2023-12-18

**Authors:** Maia E. VanDyke, Allen F. Morey, Karl Coutinho, Kaiser J. Robertson, Richard D'Anna, Kent Chevli, Christopher H. Cantrill, Michael J. Ehlert, Alexis E. Te, Jeffrey Dann, Jessica M. DeLong, Ramón Virasoro, Judith C. Hagedorn, Richard Levin, Euclid DeSouza, David DiMarco, Brad A. Erickson, Carl Olsson, Sean P. Elliott

**Affiliations:** ^1^ UT Southwestern Medical Center Dallas Texas USA; ^2^ New Jersey Urology LLC Millburn New Jersey USA; ^3^ Chesapeake Urology Hanover Maryland USA; ^4^ Arkansas Urology Little Rock Arkansas USA; ^5^ Western New York Urology Associates Cheektowaga New York USA; ^6^ Urology San Antonio San Antonio Texas USA; ^7^ Metro Urology, a division of Minnesota Urology Woodbury Minnesota USA; ^8^ Weill Cornell Medicine New York New York USA; ^9^ Advanced Urology Institute Daytona Beach Florida USA; ^10^ MultiCare Urology Puyallup Washington USA; ^11^ Surgical Services, Section of Urology VA Eastern Colorado Health Care System Aurora Colorado USA; ^12^ University of Washington Seattle Washington USA; ^13^ Chesapeake Urology Research Associates Annapolis Maryland USA; ^14^ Adult and Pediatric Urology PC Omaha Nebraska USA; ^15^ Oregon Urology Institute Springfield Oregon USA; ^16^ University of Iowa Hospitals and Clinics Iowa City Iowa USA; ^17^ Integrated Medical Professionals New York New York USA; ^18^ University of Minnesota Minneapolis Minnesota USA

**Keywords:** anterior urethral strictures, bladder outlet obstruction, lower urinary tract symptoms, urethral dilation, urethral stricture

## Abstract

**Objective:**

The aim of this study is to report the updated 2‐year results of the intervention arm of the ROBUST III randomized trial evaluating the safety and efficacy of the Optilume drug‐coated balloon (DCB) versus standard endoscopic management of recurrent male anterior urethral stricture.

**Materials and Methods:**

Eligible patients included men with recurrent anterior urethral stricture ≤3 cm in length and ≤12Fr in diameter, International Prostate Symptom Score (IPSS) ≥11 and peak flow rate (Qmax) <15 mL/s. Patients were randomized to treatment with the Optilume DCB or standard‐of‐care endoscopic management. Primary efficacy endpoints measured at 2 years included freedom from re‐intervention and changes in IPSS, Qmax and post‐void residual (PVR). Secondary endpoint was impact on sexual function using the International Index of Erectile Function (IIEF). Primary safety endpoint was freedom from serious procedure‐ or device‐related adverse events (AEs).

**Results:**

A total of 127 patients enrolled at 22 sites in the United States and Canada (48 randomized to standard‐of‐care dilation and 79 to DCB dilation). Seventy‐five patients in the DCB arm entered the open‐label phase after 6 months. Participants averaged 3.2 prior endoscopic interventions (range 2–10); most (89.9%) had bulbar strictures with an average stricture length of 1.63 cm (SD 0.76). Significant improvements in IPSS, average Qmax and PVR were maintained at 2 years. Freedom from repeat intervention was significantly higher in the Optilume DCB arm at 2 years versus the Control arm at 1 year (77.8% vs. 23.6%, *p* < 0.001). During the follow‐up period, there were 15 treatment failures and two non‐study‐related deaths. Treatment‐related AEs were rare and generally self‐limited (haematuria, dysuria and urinary tract infection).

**Conclusion:**

The Optilume DCB shows sustained improvement in both objective and subjective voiding parameters at 2‐year follow‐up. Optilume appears to provide a safe and effective endoscopic treatment alternative for short recurrent anterior urethral strictures among men who wish to avoid or delay formal urethroplasty.

## 1 INTRODUCTION

For recurrent anterior urethral stricture disease, urethroplasty is the guideline‐recommended gold standard treatment after failed endoscopic management.^1^ Even so, most urologists do not perform urethroplasty and are more likely to undertake endoscopic management despite literature confirming the superiority of the former.^2^, ^3^, ^4^ Multiple alternatives have been proposed to urethroplasty in the setting of recurrent stricture, including mechanical stents and injectable agents.^5^, ^6^, ^7^

Officially approved by the FDA in December 2021, the Optilume® drug‐coated balloon (DCB) (Urotronic, Plymouth, MN) provides a new alternative for men who do not wish to repeat standard endoscopic management but are also not interested in urethroplasty. In addition to providing coaxial dilation of the urethral lumen, the DCB locally delivers paclitaxel, an antimitotic agent that inhibits cell proliferation and has been used routinely for decades by interventional cardiologists during coronary angioplasty.^8^

The 1‐year results of the prospective, multicentre, randomized controlled ROBUST III trial demonstrated promising safety and efficacy of the Optilume system for management of recurrent anterior urethral stricture.^9^ Anatomic success at 6 months (the ability to pass a 16Fr flexible cystoscope or 14Fr catheter) was nearly three times higher in those patients in the Optilume DCB arm compared to those in the Control arm. We now report the updated 2‐year safety and efficacy outcomes of the DCB cohort of the ROBUST III trial.

## 2 METHODS

### 2.1 Trial design and oversight

The ROBUST III study is a prospective, multicentre, single blind, randomized controlled study performed to evaluate the safety and efficacy of the Optilume DCB for the treatment of male anterior urethral stricture. Ethics committee approval was received for all participating sites. The study was registered on ClinicalTrials.gov (NCT03499964). An independent data monitoring committee provided safety oversight and a clinical events committee adjudicated adverse events.

### 2.2 Patient population

Adult men with an anterior urethral stricture ≤12Fr and ≤3 cm in length measured by urethrogram, at least two prior endoscopic treatments, International Prostate Symptom Score (IPSS) ≥11 and peak urinary flow rate (Qmax) <15 mL/s were considered for participation in the study. Key criteria for exclusion were prior urethroplasty, hypospadias repair, lichen sclerosis or unresolved confounding aetiologies such as bladder neck contracture or benign prostatic hyperplasia. Written informed consent was obtained from all participants prior to enrolment.

### 2.3 Intervention and follow‐up

Participants were randomized 2:1 to receive treatment with the Optilume DCB or endoscopic management (standard of care). Post‐procedure follow‐up for all participants was performed at Foley removal (2–5 days), 30 days, 3 months, 6 months and 1 year. Randomized participants remained blinded to treatment group assignments through 6 months, after which the open‐label phase was initiated. For the DCB cohort, annual follow‐up continues through 5 years. Required study follow‐up for the standard‐of‐care group of subjects has been completed through 1 year and reported previously; this group was not followed past 1 year.^9^

Pre‐dilation of the stricture to a minimum calibre of 20Fr with an uncoated balloon or DVIU was performed prior to treatment with the Optilume DCB. DCBs were available in diameters of 18–36Fr and lengths of 3 and 5 cm. Balloon size selection was based on lumen diameter and stricture length to allow for 0.5–1 cm overlap of normal tissue on both ends of the stricture. The balloon was inflated to rated burst pressure for a minimum of 5 min followed by insertion of a 12–14Fr Foley catheter.

### 2.4 Endpoints and statistical analysis

Efficacy and safety analyses are reported for all participants randomized to the Optilume DCB group. Anatomical success was defined as urethral lumen of 14Fr or greater by calibration or cystoscopy at 6 months and has been reported on previously.^9^ Primary endpoints assessed during the 2‐year follow‐up period included freedom from re‐intervention and changes in IPSS, Qmax and post‐void residual (PVR). Secondary endpoint was impact on sexual function using the International Index of Erectile Function (IIEF) questionnaire. Primary safety endpoint was freedom from serious procedure‐ or device‐related adverse events (AEs).

Efficacy outcomes evaluated at baseline and each follow‐up visit included IPSS, quality of life (QoL), Qmax and PVR. Freedom from repeat intervention was determined via Kaplan–Meier analysis and used a log‐rank test for comparison of the rate in the Optilume DCB group through 2 years to the rate in the Control group through 1 year. Participants were right censored at the time of their last visit or at the close of the 2‐year visit window (790 days), whichever was earlier. Subgroup analyses for IPSS and Qmax were performed based on number of prior dilations (<5 vs. ≥5) and stricture length (<2 cm vs. ≥2 cm).Change from baseline to 2 years follow‐up for IPSS and Qmax were compared using an ANCOVA model adjusted for baseline values. Subgroup analysis was also performed based on stricture characteristics (stricture length, anatomic location, aetiology, radiation history and number of prior interventions) using Kaplan–Meier point estimates for freedom from reintervention. Impact on sexual function was evaluated using the International Index of Erectile Function (IIEF) questionnaire. Safety was assessed by the rate and types of reported adverse events (AEs).

Descriptive statistics were used to summarize results. A failure carried forward imputation approach was used for efficacy analyses, in which participants who were considered treatment failures (i.e., underwent repeat intervention) were assigned their worst observed value for each efficacy variable (IPSS, Qmax and PVR) for visits after repeat intervention was received. Comparisons to baseline were evaluated with a paired *t*‐test, while comparison between subgroups was evaluated with an unpaired *t*‐test. The required sample size was based on the randomized portion of the study. There were no additional sample size requirements associated with long‐term follow‐up. Significance was evaluated at the 0.05 level with no adjustments for multiplicity. Statistical analyses were performed using SAS 9.4 (SAS Institute, Cary, NC, USA).

## 3 RESULTS

The study enrolled 141 participants at 22 investigational sites in the United States and Canada between October 2018 and December 2020. Of these, 127 were randomized (79 to Optilume DCB and 48 to standard of care) and 14 participated in a pharmacokinetic (PK) sub‐study. Pharmacokinetic results have been previously reported elsewhere.^9^ All 79 men randomized to the Optilume DCB group were treated with the device.

**FIGURE 1:**
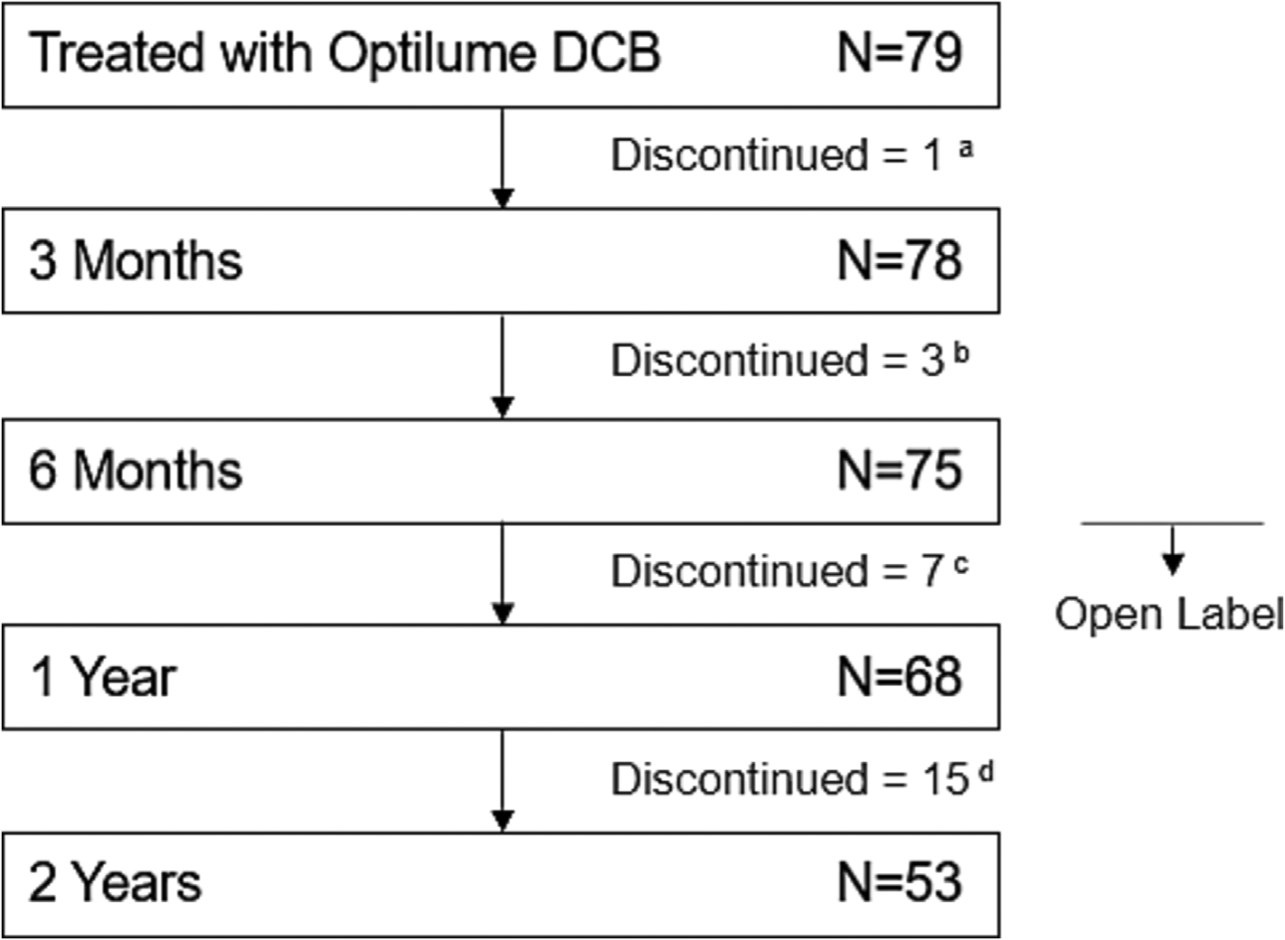
Participant timeline. Reasons for discontinuation between visits are as follows: ^a^treatment failure (1); ^b^adverse event (1: urethritis and recurrent stricture, considered a treatment failure), withdrew consent (1), death (1: intestinal infarction); ^c^treatment failure (5), withdrew consent (1), lost to follow‐up (1); ^d^treatment failure (8), withdrew consent (3), investigator discretion (2), lost to follow‐up (1), death (1: lung cancer).

On average, men treated with the Optilume DCB were 58 years old (range 25–87) and had 3.2 prior endoscopic treatments (range 2–10) at the time of enrolment. Most had bulbar strictures (89.9%) with an average length of 1.63 cm (SD 0.76). Stricture aetiology has previously been reported and was similar between the control and Optilume DCB groups with idiopathic strictures being the most common, followed by iatrogenic and traumatic causes.^9^ Strictures were pre‐dilated with an uncoated balloon (72 subjects), DVIU (3) or both (4). DCB diameters used for treatment were most commonly 30Fr (70 subjects), 24Fr (6) or 36Fr (3). A total of 75 participants entered the open label phase beginning after the 6‐month visit (Figure 1). There were 30 discontinuations prior to 2 years including 15 treatment failures and two non‐study‐related deaths (one each due to intestinal infarction and lung cancer).

### 3.1 Efficacy

**FIGURE 2:**
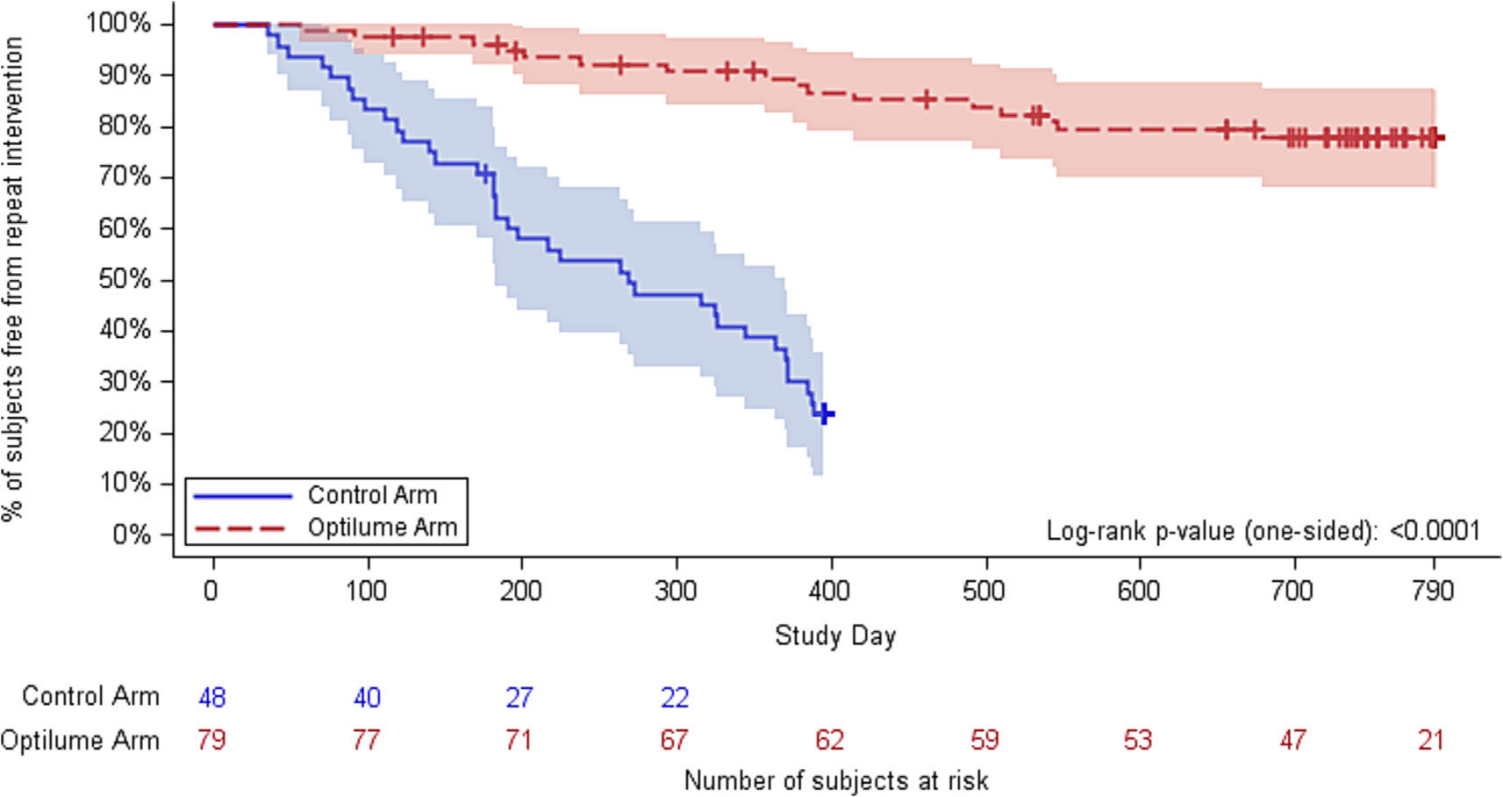
Kaplan–Meier curve for freedom from repeat intervention.

Significant improvements were maintained in IPSS and Qmax for subjects treated with the Optilume DCB over the 2‐year study interval. The average IPSS improved from 22.0 at baseline to 9.0 at 1 year and 10.1 at 2 years (*p* < 0.001; Table 1). At 2 years, 61% (38/62) of the participants experienced an IPSS improvement of at least 30% without repeat intervention. Average Qmax improved from 7.6 mL/s at baseline to 15.5 at 1 year and 12.6 at 2 years (*p* = 0.003). The Kaplan–Meier estimate for freedom from repeat intervention was significantly greater in the Optilume DCB group at 2 years (77.8%) compared to the Control group at 1 year (23.6%), yielding a difference between groups of 54.2% (*p* < 0.0001, 95% CI 38.7%–69.7%; Figure 2).

**TABLE 1:** Summary of outcome measures through 2 years for the Optilume DCB group.

Measure	Baseline	3‐Month	6‐Month	1‐Year	2‐Year
IPSS					
*n*	79	75	71	67	62
Mean ± SD	22.0 ± 6.8	7.4 ± 5.8^a^	8.3 ± 6.2^a^	9.0 ± 7.1^a^	10.1 ± 6.7^a^
IPSS QoL					
*n*	79	75	71	67	62
Mean ± SD	4.5 ± 1.3	1.5 ± 1.4^a^	1.7 ± 1.3^a^	1.9 ± 1.5^a^	2.1 ± 1.3^a^
Qmax (mL/s)					
*n*	78	71	67	65	58
Mean ± SD	7.6 ± 3.4	18.6 ± 10.9^a^	16.6 ± 8.9^a^	15.5 ± 9.0^a^	12.6 ± 7.6^a^
PVR (mL)					
*n*	77	70	67	66	59
Mean ± SD	109.8 ± 116.9	103.4 ± 134.4	73.1 ± 117.7	94.6 ± 121.8	91.9 ± 105.8
IIEF EF^b^					
*n*	48	39	40	30	21
Mean ± SD	20.8 ± 8.8	23.2 ± 8.0	23.0 ± 8.4	24.1 ± 7.4	24.2 ± 7.7

^a^ Statistically significant improvement from baseline when analysed with a paired *t*‐test.

^b^ Only subjects that were sexually active at baseline are included in this assessment.

Clinically relevant subgroups were evaluated to determine if outcomes differed for those subjects with ≥5 prior dilations and those with stricture lengths ≥2 cm. Similar to the overall cohort, sustained improvement in IPSS and Qmax through 2 years was observed for both subgroups (Table 2). The average IPSS improved from 22.4 at baseline to 9.6 at 2 years for subjects with <5 prior dilations and from 20.0 to 12.5 for subjects with ≥5 prior dilations. Subjects with stricture length <2 cm had an average IPSS of 21.2 at baseline that improved to 10.7 at 2 years, and subjects with stricture length ≥2 cm had the average IPSS improve from 23.0 to 9.6. No statistically significant differences were noted in these subgroups for outcomes at 2 years.

**TABLE 2:** IPSS and Qmax by subgroup for the Optilume DCB group.

Measure	Subgroup	Baseline	2 years	2‐year change from baseline	Difference in change from baseline	*p*‐value
Results by number of prior dilations						
IPSS	<5 prior dilations (*N* = 67)	22.4 ± 6.7 (67)	9.6 ± 6.3 (51)	−13.1 ± 9.2 (51)	−2.8 (−7.3, 1.6)	0.2088
	≥5 prior dilations (*N* = 12)	20.0 ± 7.0 (12)	12.5 ± 8.2 (11)	−8.2 ± 11.4 (11)		
Qmax (mL/s)	<5 prior dilations (*N* = 67)	7.4 ± 3.4 (66)	12.0 ± 4.9 (48)	4.3 ± 5.7 (48)	−3.5 (−8.9, 1.9)	0.1966
	≥5 prior dilations (*N* = 12)	9.0 ± 3.3 (12)	15.4 ± 15.2 (10)	6.1 ± 16.7 (10)		
Results by stricture length						
IPSS	<2 cm length (*N* = 42)	21.2 ± 7.2 (42)	10.7 ± 6.2 (33)	−11.2 ± 10.3 (33)	1.1 (−2.4, 4.6)	0.5327
	≥2 cm length (*N* = 36)	23.0 ± 6.3 (36)	9.6 ± 7.3 (28)	−13.3 ± 9.1 (28)		
Qmax (mL/s)	<2 cm length (*N* = 42)	7.9 ± 3.5 (41)	12.6 ± 9.2 (33)	4.4 ± 9.8 (33)	0.0 (−4.2, 4.3)	0.9855
	≥2 cm length (*N* = 36)	7.1 ± 3.3 (36)	12.6 ± 5.0 (24)	5.1 ± 6.5 (24)		

*Note*: Mean ± standard deviation (number of subjects) shown. Differences in change, 95% CIs, and *p*‐values were calculated based on an ANCOVA model adjusted for baseline values.

### 3.2 Safety

The most frequently reported AEs related to treatment were haematuria (13.9%), mild dysuria (6.3%) and urinary tract infection (6.3%). Most haematuria events had onset within 30 days of the procedure (9/12), were mild according to the Common Terminology Criteria for Adverse Events (11/12) and resolved within 30 days (10/12). Treatment‐related serious AEs were minimal and included one event each of aspiration/aspiration pneumonia and urinary tract infection in each arm. Sexual function was preserved through 2 years after treatment with the Optilume DCB based on the IIEF erectile function (EF) domain score (Table 1).

## 4 DISCUSSION

The 2‐year results of the ROBUST III trial further illustrate that the Optilume paclitaxel‐coated balloon is a safe and effective endoscopic therapy for the management of recurrent anterior urethral strictures ≤3 cm in length. Roughly three‐quarters of the Optilume DCB patients remained free from repeat intervention—three times higher than the 1‐year results of the Control group. The improvements in IPSS, Qmax and PVR previously reported at the 1‐year point were also maintained across the extended follow‐up period. The Optilume DCB thus represents a novel and promising alternative in the treatment paradigm of recurrent anterior urethral stricture for those patients who are not healthy enough to undergo a formal urethroplasty—or for those who simply prefer a less invasive option.

### 4.1 Efficacy

Definitions of success after treatment for urethral stricture disease vary widely, which makes it difficult to compare success rates across studies.^10^ Common definitions include cystoscopic patency, freedom from reintervention, patient‐reported outcome measures (PROMs) and changes on uroflowmetry, with the probability of ‘success’ varying widely depending on the definition chosen.^10^ In this trial, several measures of success were examined in order to provide a more comprehensive evaluation. When compared to the 1‐year results of the Control arm, Optilume continued to significantly outperform standard endoscopic management with regard to freedom from re‐intervention at 2 years. Improvements in IPSS, Qmax and PVR were also sustained over a 2‐year period.

Patients at higher risk for recurrence (stricture length ≥2 cm or history of ≥5 prior dilations) also saw significant improvements in IPSS and Qmax, which were sustained over 2 years. In fact, those at higher risk of recurrence had similar improvements in both parameters when compared to those at lower risk of recurrence (*p* > 0.05). Other at‐risk groups (such as those with history of radiation) were not evaluated given their low numbers in this study population. Kaplan–Meier point estimates were used to evaluate the probability of freedom from reintervention based on stricture characteristics and are shown in Table 3.

**TABLE 3:** Kaplan–Meier point estimates for freedom from reintervention at 1 and 2 years for the Optilume DCB group based on stricture characteristics.

Subgroup	1‐year point estimate (95% CI)	2‐year point estimate (95% CI)
Overall (*n* = 79)	86.9% (79.4%, 94.5%)	78.5% (69.2%, 87.9%)
Stricture length		
<2 cm (*n* = 42)	83.0% (71.5%, 94.5%)	72.6% (58.7%, 86.5%)
≥2 cm (*n* = 36)	91.3% (81.9%, 100.0%)	85.3% (73.4%, 97.2%)
Anatomic location		
Bulbar (*n* = 71)	88.3% (80.6%, 95.9%)	82.0% (72.7%, 91.2%)
Penile (*n* = 8)	75.0% (45.0%, 100.0%)	46.9% (10.3%, 83.4%)
Stricture aetiology		
Iatrogenic (*n* = 21)	75.9% (57.5%, 94.3%)	65.4% (44.5%, 86.3%)
Idiopathic (*n* = 43)	87.8% (78.1%, 97.9%)	79.9% (67.4%, 92.4%)
Traumatic (*n* = 14)	100.0% (−, −)	92.3% (77.8%, 100.0%)
Prior radiation (*n* = 9)	76.2% (47.2%, 100.0%)	76.2% (47.2%, 100.0%)
No. prior treatments		
<5 (*n* = 67)	87.6% (79.2%, 95.6%)	79.2% (69.1%, 89.3%)
≥5 (*n* = 12)	83.3% (62.2%, 100.0%)	75.0% (50.5%, 99.5%)

### 4.2 Urethroplasty versus Optilume

Although urethroplasty has been shown to outperform repeated endoscopic management of recurrent urethral stricture, endoscopic management is still used more frequently than urethroplasty for various reasons.^2^, ^4^, ^11^, ^12^ Urethroplasty tends to be performed at high volume academic centres, while community urologists are more likely to opt for endoscopic management.^4^, ^13^, ^14^ Second, urethroplasty remains a more extensive surgery, resulting in additional morbidity including pain, bleeding risk and prolonged recovery time. Third, urethroplasty requires more prolonged catheter duration post‐operatively, which is a deterrent for many patients given the impact on quality of life.^15^, ^16^ Lastly, some patients are concerned about the risk of sexual dysfunction after urethroplasty, although most patients do recover erectile function over time.^17^, ^18^, ^19^

Optilume balloon dilation is a straightforward procedure that provides a viable alternative to urethroplasty. It is both faster and less invasive than urethroplasty and can be performed in the clinic setting via a flexible cystoscope, which may provide additional benefits. Patients may return to normal activity levels—with the exception of sexual activity—within days after the procedure, allowing earlier return to work and a shorter catheter duration. Patients are counselled to abstain from sexual activity for 14 days post‐procedure and utilize effective contraceptive for at least 6 months post‐procedure due to the presence of small amounts of paclitaxel in semen after treatment.^9^

### 4.3 Dilation considerations

Methods for mechanical disruption of urethral stricture include balloon dilation, DVIU and sequential dilation using urethral sounds.^20^, ^21^ Although some surgeons prefer DVIU given the ability to directly visualize the stricture at time of dilation, both balloon dilation and rigid dilators have been shown to outperform DVIU with respect to long‐term retreatment rate.^21^, ^22^, ^23^ Concerns regarding poor placement of the balloon during dilation may be obviated either by performing balloon dilation under direct vision or by using intra‐operative fluoroscopy for stricture localization.^21^, ^24^, ^25^ In the ROBUST III cohort, there was no significant difference in retreatment rates in either arm based on dilation/pre‐dilation method. However, uncoated balloon dilation was by far the preferred method of dilation in both arms.

In the DCB arm, surgeons were given the choice of which balloon to use for pre‐dilation. While Laborie offers a bundle including both the DCB and a low‐pressure non‐coated balloon (rated burst pressure 10 atm), the most commonly used balloon dilators on the market are so‐called ‘high‐pressure’ balloons with rated burst pressures of 20 atm or greater (such as the UroMax™, Boston Scientific Corporation, USA). No direct comparison studies currently exist between low‐ and high‐pressure balloons, although anecdotally multiple authors on this study have seen occasional strictures resistant to dilation with a low‐pressure balloon.

Surgeons were also given the discretion to select between 18–36Fr balloon diameters, with a goal of selecting a balloon that is slightly greater than the diameter of the adjacent, healthy urethra.^25^ Most standard‐of‐care subjects treated with a balloon in the ROBUST III control arm were treated with a 24Fr balloon, while in the treatment arm, the most used DCB size was 30F (89%).^9^ Although this raises the question on whether balloon diameter could be a confounder, post‐treatment urethrogram showed similar urethral diameter in both groups. Moreover, a previously published subset analysis of those treated with 30Fr balloons showed similar findings to the overall analysis.^9^ The sustained results seen at 2 years also make it less likely that the treatment difference at 1 year was due to balloon size. Interestingly, the most common balloon diameters cited in the literature are 21–24Fr.^23^, ^26^ This is the largest reported study using a 30Fr balloon for urethral dilation, and our results support its safety and efficacy in this setting.

### 4.4 Safety

The previously published 1‐year results from ROBUST III showed higher rates of haematuria and dysuria in the Optilume group compared to the Control group.^9^ However, these AEs were predominantly mild and self‐limited. Serious AEs included one event each of aspiration pneumonia and urinary tract infection in each arm. No additional treatment‐related AEs were reported in the Optilume group after the 12‐month timepoint. Sexual function, as determined by the IIEF erectile function domain score, was preserved through 2 years of post‐treatment monitoring.

As an anti‐mitotic agent, paclitaxel has been extensively studied in the cardiovascular field where it is used for angioplasty with drug eluting stents and drug coated balloons.^27^, ^28^ Unlike mitomycin C which is a cytotoxic agent, paclitaxel works cytostatically, inhibiting microtubule function and mitosis.^29^ Recently, the efficacy of injectable mitomycin C has been called into question for the treatment of stricture disease, given poor long‐term efficacy and a 7% risk of serious adverse events.^7^ By contrast, serious AEs were rare in our 2‐year data and not significantly different between the two treatment arms.

### 4.5 Limitations

Our study is not without limitations; first, this was a single‐blind study where the surgeons were not blinded to the type of treatment administered. Patients were unblinded after 6 months, and this could impact how they completed questionnaires such as the IPSS and their interest in re‐treatment for recurrent symptoms. However, this would not impact the Qmax or PVR results reported here. Moreover, surgeons were permitted to choose the method of pre‐dilation (DVIU, sequential dilation with urethral sounds or balloon dilation) as well as the method of dilation for the Control arm. This lack of standardization led to more heterogeneity in the population, which theoretically hinders direct comparisons between arms. However, there was no statistically significant difference in outcome between these methods in either arm upon subanalysis. It is also notable that the majority of patients in the control arm were dilated to 24Fr while the majority of those in the treatment arm were dilated to 30Fr. Certainly, it would be ideal for future study design to include a standardized dilation to 30Fr in both groups via a consistent method (i.e., balloon dilation) in order to achieve a more straightforward comparison between populations.

We also acknowledge that pre‐dilation in the DCB arm introduces bias, as patients in this arm underwent two dilations compared to just one in the Control arm. It also has a potential cost impact; the upfront costs are clearly higher when two dilations are performed. However, an independent cost analysis indicates that the improved freedom from reintervention in the DCB arm may well lead to cost savings when compared to standard endoscopic management when factoring in the need for retreatment in patients over a 5‐year time horizon. Additionally, pre‐dilation allows for full assessment of the proximal urethra prior to deployment of the DCB to ensure there are no additional strictures, and of the bladder to ensure no lesions or other concerning findings.

As is the nature of prospective studies, not all patients initially enrolled in the Optilume DCB arm were followed to completion of the 2‐year endpoint. Some of these patients withdrew consent, while others were lost to follow‐up or expired from unrelated causes. Additional multi‐institutional studies over the coming years will help to confirm these results, as will the long‐term follow‐up data from this cohort, which is planned to extend to 5 years.

Our study was not powered to detect differences in success between certain subgroups, including those with history of radiation. At this time, these results cannot be generalized to the post‐radiation setting, although certainly future studies will be imperative to study this difficult population. Moreover, some strictures with poor prognostic features (i.e., those due to lichen sclerosis) were excluded from this study. Thus, we cannot draw any conclusions as to the efficacy of the Optilume DCB system in these populations. Lastly, we do not yet know if or how prior Optilume may affect future urethroplasty in those men who progressed to treatment failure, or whether repeat Optilume dilation could present a viable option in this setting.

## 5 CONCLUSIONS

The Optilume DCB delivers improvements in both objective and subjective voiding parameters that are maintained over a 2‐year follow‐up period. Optilume appears to offer a safe and superior alternative to standard endoscopic management for treating short recurrent anterior urethral strictures and a viable treatment alternative for those men who wish to avoid or delay formal urethroplasty.

## AUTHOR CONTRIBUTIONS

**Maia E. VanDyke:** Investigation, analysis, manuscript drafting and preparation. **Allen F. Morey:** Investigation; analysis; manuscript drafting and preparation. **Karl Coutinho:** Investigation; manuscript preparation. **Kaiser J. Robertson:** Investigation; manuscript preparation. **Richard D'Anna:** Investigation; manuscript preparation. **Kent Chevli:** Investigation; manuscript preparation. **Christopher H. Cantrill:** Investigation; manuscript preparation. **Michael J. Ehlert:** Investigation; manuscript preparation. **Alexis E. Te:** Investigation; manuscript preparation. **Jeffrey Dann:** Investigation; manuscript preparation. **Jessica M. DeLong:** Investigation; manuscript preparation. **Ramón Virasoro:** Investigation; manuscript preparation. **Judith C. Hagedorn:** Investigation; manuscript preparation. **Richard Levin:** Investigation; manuscript preparation. **Euclid DeSouza:** Investigation; manuscript preparation. **David DiMarco:** Investigation; manuscript preparation. **Brad A. Erickson:** Investigation; manuscript preparation. **Carl Olsson:** Investigation; manuscript preparation. **Sean P. Elliott:** Conceptualization; methodology; investigation; analysis; manuscript preparation.

## CONFLICT OF INTEREST STATEMENT

VanDyke: None. Morey: Coloplast, Boston Scientific. Coutinho: Urotronic. Robertson: None. D'Anna: None. Chevli: None. Cantrill: None. Ehlert: Coloplast, Medtronic, Valencia Technologies. Te: Biobot, Boston Scientific, Meditate, Procept, Urotronic, Olympus, Zenflo. Dann: Urotronic. DeLong: Laborie, Urovant. Virasoro: Laborie. Hagedorn: None. Levin: Boston Scientific. DeSouza: None. DiMarco: None. Erickson: Boston Scientific. Olsson: Exilixis Corp. Elliott: Boston Scientific, Laborie, Percuvision, Urotronic.

## References

[1] Wessells H , Angermeier KW , Elliott S , Gonzalez CM , Kodama R , Peterson AC , et al. Male urethral stricture: American Urological Association guideline. J Urol. 2017;197(1):182–190. 10.1016/j.juro.2016.07.087 27497791

[2] Bullock TL , Brandes SB . Adult anterior urethral strictures: a national practice patterns survey of board certified urologists in the United States. J Urol. 2007;177(2):685–690. 10.1016/j.juro.2006.09.052 17222657

[3] JT Anger , Buckley JC , Santucci RA , Elliott SP , Saigal CS , Urologic Diseases in America Project . Trends in stricture management among male Medicare beneficiaries: underuse of urethroplasty? Urology. 2011;77(2):481–485. 10.1016/j.urology.2010.05.055 21168194 PMC3320109

[4] Liu JS , Hofer MD , Oberlin DT , Milose J , Flury SC , Morey AF , et al. Practice patterns in the treatment of urethral stricture among American urologists: a paradigm change? Urology. 2015;86(4):830–834. 10.1016/j.urology.2015.07.020 26216643

[5] Palminteri E , Gacci M , Berdondini E , Poluzzi M , Franco G , Gentile V . Management of urethral stent failure for recurrent anterior urethral strictures. Eur Urol. 2010;57(4):615–621. 10.1016/j.eururo.2009.11.038 20018439

[6] Pranata FH , Hidayatullah F , Kloping YP , Rahman ZA , Rizaldi F , Soebadi DM . The efficacy and safety of mitomycin C intra urethral injection to prevent recurrent urethral stricture: a systematic review and meta‐analysis. Ann Med Surg (Lond). 2022;77:103576. 10.1016/j.amsu.2022.103576 35638056 PMC9142380

[7] Redshaw JD , Broghammer JA , Smith TG , Voelzke BB , Erickson BA , McClung CD , et al. Intralesional injection of mitomycin C at transurethral incision of bladder neck contracture may offer limited benefit: TURNS Study Group. J Urol. 2015;193(2):587–592. 10.1016/j.juro.2014.08.104 25200807 PMC4307389

[8] Axel DI , Kunert W , Göggelmann C , Oberhoff M , Herdeg C , Küttner A , et al. Paclitaxel inhibits arterial smooth muscle cell proliferation and migration in vitro and in vivo using local drug delivery. Circulation. 1997;96(2):636–645. 10.1161/01.CIR.96.2.636 9244237

[9] Elliott SP , Coutinho K , Robertson KJ , D'Anna R , Chevli K , Carrier S , et al. One‐year results for the ROBUST III randomized controlled trial evaluating the Optilume® drug‐coated balloon for anterior urethral strictures. J Urol. 2022;207(4):866–875. 10.1097/JU.0000000000002346 34854748 PMC12721643

[10] Anderson KT , Vanni AJ , Erickson BA , Myers JB , Voelzke B , Breyer BN , et al. Defining success after anterior urethroplasty: an argument for a universal definition and surveillance protocol. J Urol. 2022;208(1):135–143. 10.1097/JU.0000000000002501 35239415

[11] Heyns C , Steenkamp J , De Kock M , Whitaker P . Treatment of male urethral strictures: is repeated dilation or internal urethrotomy useful? J Urol. 1998;160(2):356–358. 10.1016/S0022-5347(01)62894-5 9679876

[12] Zaid UB , Lavien G , Peterson AC . Management of the recurrent male urethral stricture. Curr Urol Rep. 2016;17(4):33. 10.1007/s11934-016-0588-0 26902627

[13] Burks FN , Salmon SA , Smith AC , Santucci RA . Urethroplasty: a geographic disparity in care. J Urol. 2012;187(6):2124–2127. 10.1016/j.juro.2012.01.078 22503011

[14] Moynihan MJ , Voelzke B , Myers J , Breyer BN , Erickson B , Elliott SP , et al. Endoscopic treatments prior to urethroplasty: trends in management of urethral stricture disease. BMC Urol. 2020;20(1):68. 10.1186/s12894-020-00638-x 32534592 PMC7293125

[15] Jang EB , Hong SH , Kim KS , Park SY , Kim YT , Yoon YE , et al. Catheter‐related bladder discomfort: how can we manage it? Int Neurourol J. 2020;24(4):324–331. 10.5213/inj.2040108.054 33401353 PMC7788325

[16] Chan SP , Tan GWL , Ho CK . Is transurethral catheterisation the ideal method of bladder drainage? A survey of patient satisfaction with indwelling transurethral urinary catheters. Asian J Surg. 2010;33(1):31–36. 10.1016/S1015-9584(10)60006-1 20497880

[17] Feng C , Xu Y‐M , Barbagli G , Lazzeri M , Tang CY , Fu Q , et al. The relationship between erectile dysfunction and open urethroplasty: a systematic review and meta‐analysis. J Sex Med. 2013;10(8):2060–2068. 10.1111/jsm.12181 23656595

[18] Blaschko SD , Sanford MT , Cinman NM , McAninch JW , Breyer BN . D e novo erectile dysfunction after anterior urethroplasty: a systematic review and meta‐analysis. BJU Int. 2013;112(5):655–663. 10.1111/j.1464-410X.2012.11741.x 23924424 PMC3740455

[19] Baumgarten AS , Hudak SJ , Morey AF . Erectile dysfunction after urethroplasty: is the risk overstated? J Sex Med. 2020;17(2):171–173. 10.1016/j.jsxm.2019.09.020 31680005

[20] Nomikos M , Papanikolaou S , Athanasopoulos G , Papatsoris A . The use of Amplatz renal dilators in the minimally invasive management of complex urethral strictures. Central Eur J Urol. 2017;70:301. 10.5173/ceju.2017.1218 PMC5656362 29104795

[21] Kumano Y , Kawahara T , Mochizuki T , Takamoto D , Takeshima T , Kuroda S , et al. Management of urethral stricture: high‐pressure balloon dilation versus optical internal urethrotomy. LUTS: Lower Urin Tract Sympt. 2019;11(2):O34–O37. 10.1111/luts.12208 29119701

[22] Karsli O , Ustuner M , Memik O , Ulukaradag E . Comparison of urethral dilation with Amplatz dilators and internal urethrotomy techniques for the treatment of urethral strictures. Urol J. 2020;17(1):68–72. 10.22037/uj.v0i0.4662 31984473

[23] Yu S‐c , Wu H‐y , Wang W , Xu LW , Ding GQ , Zhang ZG , et al. High‐pressure balloon dilation for male anterior urethral stricture: single‐center experience. J Zhejiang Univ‐SCI B. 2016;17(9):722–727. 10.1631/jzus.B1600096 27604864 PMC5018619

[24] Vyas JB , Ganpule AP , Muthu V , Sabnis RB , Desai MR . Balloon dilatation for male urethral strictures “revisited”. Urol Ann. 2013;5(4):245. 10.4103/0974-7796.120296 24311903 PMC3835981

[25] Elterman DS , Coutinho K , Hagedorn JC . How I do it: the Optilume drug‐coated balloon for urethral strictures. Can J Urol. 2020;27:10323. 32861260

[26] Beeder LA , Cook GS , Nealon SW , Badkhshan S , Sanders SC , Perito DP , et al. Long‐term experience with balloon dilation for short bulbar and membranous urethral strictures: establishing a baseline in the active drug treatment era. J Clin Med. 2022;11(11):3095. 10.3390/jcm11113095 35683482 PMC9181788

[27] Grube E , Silber S , Hauptmann KE , Mueller R , Buellesfeld L , Gerckens U , et al. TAXUS I: six‐and twelve‐month results from a randomized, double‐blind trial on a slow‐release paclitaxel‐eluting stent for de novo coronary lesions. Circulation. 2003;107(1):38–42. 10.1161/01.CIR.0000047700.58683.A1 12515740

[28] Rosenfield K , Jaff MR , White CJ , Rocha‐Singh K , Mena‐Hurtado C , Metzger DC , et al. Trial of a paclitaxel‐coated balloon for femoropopliteal artery disease. N Engl J Med. 2015;373(2):145–153. 10.1056/NEJMoa1406235 26106946

[29] Grube E , Buellesfeld L . Paclitaxel‐eluting stents. Am J Cardiovasc Drugs. 2004;4(6):355–360. 10.2165/00129784-200404060-00003 15554720

